# Effects of shielding on the induction of 53BP1 foci and micronuclei after Fe ion exposures

**DOI:** 10.1093/jrr/rrt078

**Published:** 2013-05-31

**Authors:** Wentao Hu, Hailong Pei, He Li, Nan Ding, Jinpeng He, Jufang Wang, Yoshiya Furusawa, Ryoichi Hirayama, Yoshitaka Matsumoto, Cuihua Liu, Yinghui Li, Tetsuya Kawata, Guangming Zhou

**Affiliations:** 1 Department of Space Radiobiology, Key Laboratory of Heavy Ion Radiation Biology and Medicine, Institute of Modern Physics, Chinese Academy of Sciences, 509 Nanchang Road, Lanzhou 730000, China; 2 University of Chinese Academy of Sciences, Beijing 100049, China; 3 Research Center for Charged Particle Therapy, National Institute of Radiological Sciences, Chiba 263-555, Japan; 4 State Key Laboratory of Space Medical Fundamentation and Application Astronaut Center of China, Beijing 100094, China; 5 Department of Radiology, School of Medicine, Keio University, Tokyo, 160-8582, Japan

**Keywords:** space radiation, shielding, secondary particles, survival, DNA damage

## Abstract

High atomic number and high-energy (HZE) particles in deep space are of low abundance but substantially contribute to the biological effects of space radiation. Shielding is so far the most effective way to partially protect astronauts from these highly penetrating particles. However, simulated calculations and measurements have predicted that secondary particles resulting from the shielding of cosmic rays produce a significant fraction of the total dose and dose equivalent. In this study, we investigated the biological effects of secondary radiation with two cell types, and with cells exposed in different phases of the cell cycle, by comparing the biological effects of a 200 MeV/u iron beam with a shielded beam in which the energy of the iron ion beam was decreased from 500 MeV/u to 200 MeV/u with PMMA, polyethylene (PE), or aluminum. We found that beam shielding resulted in increased induction of 53BP1 foci and micronuclei in a cell-type-dependent manner compared with the unshielded 200 MeV/u Fe ion beam. These findings provide experimental proof that the biological effects of secondary particles resulting from the interaction between HZE particles and shielding materials should be considered in shielding design.

## INTRODUCTION

Long-duration outer space missions will expose astronauts to the high-charge and -energy (HZE) ions of the Galactic Cosmic Rays (GCRs) whose ionization patterns in molecules, cells and tissues, and the resulting biological insults, are distinct from typical terrestrial radiation [1]. HZE ions belong to high linear-energy-transfer (LET) radiation, inducing more clustered DNA damage than low-LET radiation [2, 3], thus resulting in difficulties in DNA damage repair and increased genome instability that could lead to carcinogenesis [4, 5] and other severe damages [6].

Shielding, including spacecraft walls and specific shielding materials, is one of the strategies seeking to protect astronauts efficiently [7], however, the energy spectrum of GCR peaks at ∼1000 MeV/u [8], and shielding that thick is infeasible for spacecraft launch systems because of its mass, and would only partially reduce the effective GCR dose anyway [9]. Shielding is still a practical countermeasure for exposure to GCRs during space travel, and it does attenuate biological responses of space radiation [10, 11], to a varying degree dependent on shield thickness and material [12]. To fully understand and evaluate its effects and efficiency should be of significance to practical applications.

It is well known that light hydrogenated materials provide the best shielding against space radiation. Water, polyethylene, Lucite (PMMA) and aluminum are considered as common shielding materials. Hydrogenated carbon nanofibers [13], Kevlar and Nextel [14] have also been found to be promising. However, no matter what type of shielding material is used, its interaction with highly energized particles produces nuclear fragmentation, or so-called ‘secondary particles’ [15, 16]. For cosmic rays, secondary particles account for a significant fraction of the total dose [17]. A detailed mechanistic knowledge of the astronaut's irradiation conditions provides a crucial basis for shielding design [18]. Secondary effects have been reported with chromosomal aberration [12] and DNA damage assays [19]. However, the biological effects of secondary particles should be further examined for improved shielding design.

In this study, we investigated the effects of secondary particles resulting from the interaction of 500 MeV/u iron ions with shielding materials by comparing the biological effects of an iron ion beam (200 MeV/u) with the shielded beam of the same average energy (200 MeV/u) for Fe ions (produced as a higher energy beam attenuated through a certain thickness of PMMA, polyethylene (PE), or aluminum).

## MATERIALS AND METHODS

### Cell lines

Human embryonic lung normal cell line MRC-5 was obtained from RIKEN Bio Resource Center, and the human melanoma cell line 92-1 was kindly provided by Martine J. Jager and H. Monique H. Hurks (Leiden University Medical Center, Leiden, the Netherlands). MRC-5 and 92-1 cells were routinely cultured in MEM and RPMI-1640 medium (Sigma, St Louis, MO, USA), respectively, and complemented with 10% fetal bovine serum (FBS, Hyclone, Logan, UT, USA), 100 µg/ml streptomycin, and 100 units/ml penicillin in a humidified atmosphere of 5% CO_2_. The doubling time was 36 and 28 h for MRC-5 and 92-1 cells, respectively. G0 cells, G1 cells, and exponentially growing cells were obtained as previously described [20]. Briefly, 1 × 10^5^ cells plated in T25 flasks were allowed to grow for 8 days to become quiescent (G0 phase) by contact inhibition with one medium refreshment on Day 4. Then, cells were harvested, subcultured in a new flask of the same size and incubated for 6 h in fresh medium to allow them to reach G1 phase. One quarter of the G0 cells from a confluent flask was cultured in a new flask of the same size and allowed to grow for 24 h to reach the exponentially growing phase. Cell cycle methodology and molecular markers detection also proved the accuracy of the cell models [20]. For 92-1 cells, 5 × 10^5^ cells were seeded in 25 cm^2^ culture flasks and 2 days later were submitted to irradiation.

### Irradiation

**Fig. 1.**
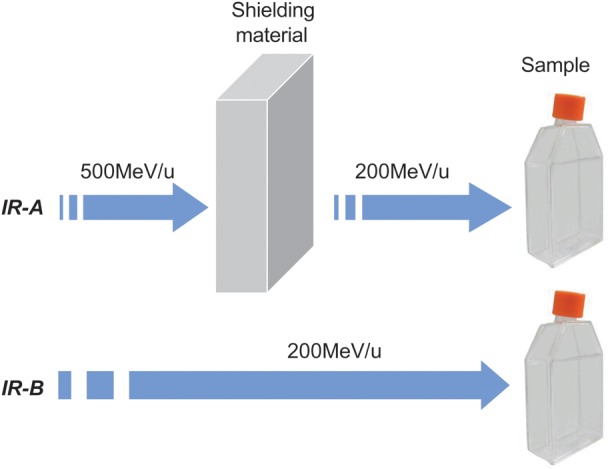
Demonstration of irradiation methods. Irradiation A (IR-A) was to use a shielding material to reduce iron ion beam energy from the primary energy of 500 MeV/u to the final mean energy at the sample of 200 MeV/u. For irradiation B (IR-B), a lower primary energy (200 MeV/u) beam was directly used to treat the cells without any shielding material.

Iron ion beams were generated by the Heavy-Ion Medical Accelerator in Chiba (HIMAC) at the National Institute of Radiological Sciences (NIRS), Chiba, Japan. The details concerning the beam characteristics of heavy ion beams, biological irradiation procedures, and dosimetry have been described elsewhere [21]. Briefly, the energy of iron ion beams at the irradiation site was obtained by comparing the calculated and measured depth–dose distribution. The irradiation doses were monitored with a small parallel-plate ionization chamber placed at the irradiation site. In order to study the effects of secondary particles, we used two irradiation strategies. As shown in Fig. 1, in the case of ‘irradiation A’ (IR-A, 500 MeV/u), shielding materials were used to reduce the beam energy to the same energy as ‘irradiation B’; in the case of ‘irradiation B’ (IR-B, 200 MeV/u), an iron ion beam with a primary energy of 200 MeV/u was directly used to treat cells. The energy listed in the text and Fig. 1 was the energy before exiting the beam window (500 MeV/u and 200 MeV/u). However, the actual energy on the samples was 420 MeV/u and 115 MeV/u, respectively, due to the scattering of the window and air. The thickness of the shield was 57.7 mm, 75.3 mm and 34.7 mm for PMMA, polyethylene and aluminum, respectively. In both irradiation methods, the samples were exposed to an iron ion beam with the same ion energy and, thus, the difference in biological effects between IR-A and IR-B was presumably induced by the secondary particles produced by the shielding. The dose rate for Fe ion exposures was about 0.9 Gy/min. X-rays were generated by a Faxitron RX-650 (Faxitron Bioptics, Lincolnshire, IL, USA), which was operated at 100 kVp 5 mA at room temperature. The dose rate was 1.3 Gy/min.

### 53BP1 foci

Irradiated cells were fixed for 10 min in 4% paraformaldehyde, permeabilized for 5 min in methanol at − 20°C, blocked for 1 h with 5% skim milk, and stained with mouse anti-53BP1 antibody (Upstate Biotechnology, Lake Placid, NY, USA) for 2 h. The bound antibody was visualized using Alexa Fluor^®^ 594 anti-mouse antibody (Molecular Probes, Eugene, OR, USA) and cell nuclei were counterstained with DAPI (PharMingen, San Jose, CA, USA). Slides were observed under a confocal laser scanning microscope (Nikon, Tokyo, Japan). The foci were identified using the NASA software 53BP1 foci in each cell were counted and at least 100 cells were scored for each sample.

### Micronucleus assay

Immediately after irradiation, cells were collected and reseeded in 12-well plates. Simultaneously, 2.5 µg/ml of cytochalasin B (Sigma, St Louis, MO, USA) was added into each well. After 36 h, cells were washed with PBS and fixed with methanol-glacial acetic acid (9:1, V/V). After staining with 150 µg/ml acridine orange, at least 500 binucleated cells for each sample were counted.

### Statistics

All experiments were independently repeated at least three times, and all data were presented as the means ± standard error. Student's *t*-tests were used for statistical analysis. Probability (*P*) values less than 0.05 were considered to be statistically significant.

## RESULTS

**Fig. 2.**
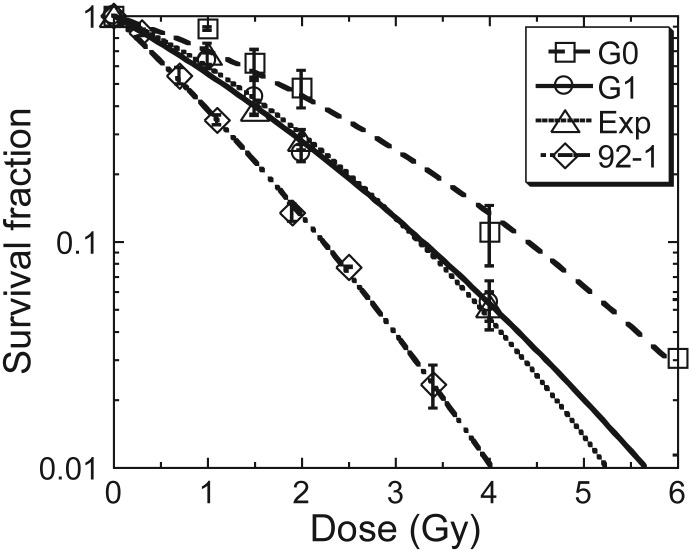
Survival curves of cells in different cycle conditions exposed to X-rays were measured with a routine colony-forming assay. Data were presented as mean ± SE. Experiments were independently repeated at least three times.

In order to investigate the biological effects of secondary particles produced by the interaction of heavy ion particles and space shielding material, we used two irradiation strategies (Fig. 1). For strategy one (IR-A), PMMA (57.7 mm), polyethylene (PE, 75.3 mm) and aluminum (34.7 mm) were used as shielding materials to reduce beam energy from the primary energy of 500 MeV/u to the final mean energy of 200 MeV/u; for strategy two (IR-B), no shielding was used so that both the primary and the final energies were 200 MeV/u. Therefore, the difference in biological effects between IR-A and IR-B were assumed to be due to the secondary particles produced by the interaction of the ion beam and the shielding materials. One cell type in three different cell cycle conditions at the time of exposure, and another cell type used in this study, showed different radiosensitivities, as measured by survival in response to X-rays (Fig. 2). Human embryonic lung normal fibroblast MRC-5 cells were cultured in three different stages, G0, G1 and the exponentially growing phase. The radiosensitivity of G1 cells was very similar to that of the exponentially growing cells. G0 MRC-5 cells were the most radioresistant among the four cell models. Human melanoma cell line 92-1 was the most sensitive [22].

**Fig. 3.**
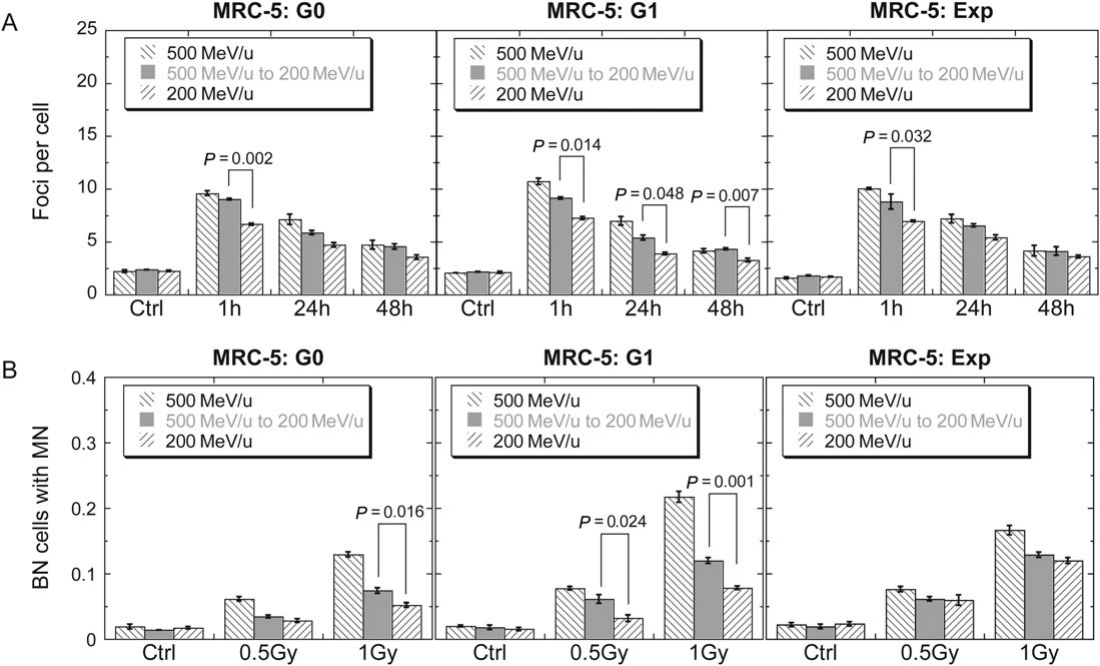
Secondary effects of PMMA on G0, G1 and exponentially growing MRC-5 cells. (**A**) Kinetics of 53BP1 foci in cells exposed to 0.5 Gy iron ions. (**B**) Binucleated cells (BN) with micronuclei (MN), which were obtained with a cytochalasin B-blocked micronucleus assay. Data were presented as mean ± SE. Experiments were independently repeated at least three times. *P*-values for comparisons between IR-A and IR-B were shown.

In order to investigate the biological effects induced by secondary particles, we measured two endpoints while using PMMA as the shielding material with MRC-5 cells in G0 and G1 phases of the cell cycle and in the exponentially growing condition at the time of exposure. First, we compared the difference in DNA damage induction and repair between IR-A (with PMMA) and IR-B (without PMMA) by 53BP1 foci quantification for a dose of 0.5 Gy at the location of the sample. As shown in Fig. 3A, in all three cell cycle conditions, significantly higher DNA damage level was induced at 1 h post IR-A exposure than at 1 h post IR-B exposure. The trend appeared to be similar at 24 and 48 h after exposure. After 48 h, a significantly higher residual DNA damage level was still present in these MRC-5 cells. In addition, we measured micronucleus frequency with a cytochalasin B-blocked micronucleus assay. As shown in Fig. 3B, IR-A appeared to induce more micronuclei than IR-B, particularly in G1 MRC-5 cells.

**Fig. 4.**
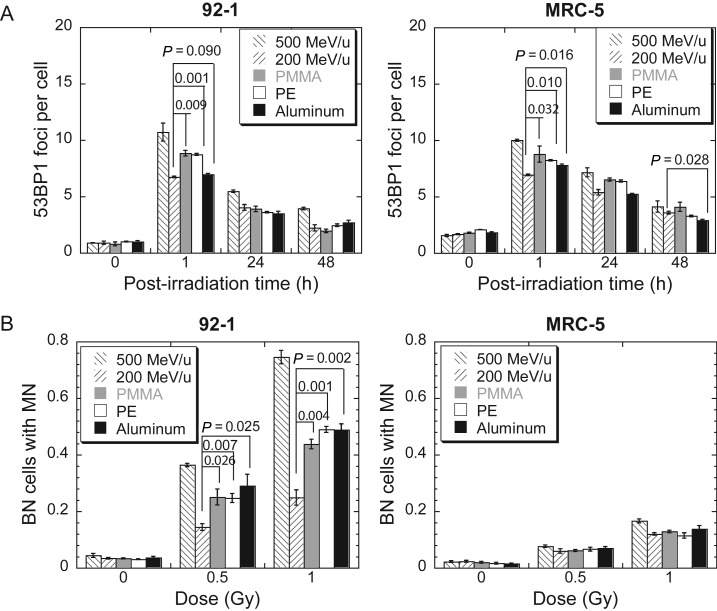
Secondary effects of three types of shielding materials: PMMA, polythylene (PE), and alumninum. (**A**) Kinetics of 53BP1 foci in 92-1 cells and exponentially growing MRC-5 cells exposed to 0.5 Gy iron ions. (**B**) Binucleated cells (BN) with micronuclei (MN), which were obtained with a cytochalasin B-blocked micronucleus assay. Data were presented as mean ± SE. Experiments were independently repeated at least three times. *P*-values for comparisons between IR-A and IR-B were shown.

In order to confirm the observed biological effects of secondary particles and compare the efficiency of various shielding materials, we conducted the 53BP1 immunofluorescence and micronucleus assay with 92-1 cells and with exponentially growing MRC-5 cells. Five schemes of irradiation were used: 500 MeV/u Fe ions, 200 MeV/u Fe ions (IR-B), and 200 MeV/u Fe ions and secondary particles after shielding of 500 MeV/u (IR-A) with PMMA (57.7 mm), polyethylene (PE, 75.3 mm) or aluminum (34.7 mm). The doses delivered to the cells were 0.5 Gy in all five cases. One hour after irradiation, 53BP1 foci were drastically induced by all five schemes of irradiation (Fig. 4A). However, strategy one (IR-A) induced many more foci than strategy two (IR-B), except when aluminum was used as a shielding material for the 92-1 cells exposure (*P* = 0.090). Fewer foci were induced in the 92-1 cells by the beam shielded with aluminum than when it was shielded with PMMA or PE (*P* = 0.011 comparing aluminum with PMMA, *P* = 0.0004 comparing aluminum with PE). No significant difference between the three shielding materials was detected for MRC-5 cells 1 h after irradiation, but 24 h later, the residual foci in the MRC-5 cells exposed to the aluminum-shielded beam were lower than those exposed to the PMMA-shielded beam (*P* = 0.008) or the PE-shielded beam (*P* = 0.002). At 48 h post-irradiation, no difference in the residual foci number between IR-A and IR-B was found except for in the MRC-5 cells exposed to the aluminum-shielded beam (*P* < 0.05). Micronuclei induced in the 92-1 cells by IR-A were significantly higher than those induced by IR-B, but for exponentially growing MRC-5 cells, no secondary effects were observed with any shielding material (Fig. 4B). For 92-1 cells, no statistical difference was observed in micronucleus induction between the shielding materials.

## DISCUSSION

Shielding is one method of protection against space radiation [12, 23], but shielding design models should be benchmarked using not only physics but also biological data [24]. In this study, we used iron ions (which contribute greatly to the dose equivalent of GCR) to expose human cells to radiation, and confirmed the biological effects of secondary particles, which should thus not be neglected in the design of spacecraft shielding. Wilson *et al*. predicted strong dependence of the attenuation of GCR-induced biological effects on the biological endpoint, the response model used, and the material composition [13]. Here we demonstrated that the biological effects of secondary particles resulting from the interaction of iron ions and shielding materials may depend on the cell type and the biological endpoints.

The biological effects of secondary particles were dependent on cell models, and cells in different cell cycle phase responded to the secondary particles differently. G0 and G1 MRC-5 cells showed significant differences in both 53BP1 foci and micronucleus induction between IR-A and IR-B (Fig. 3). Difference was also observed in foci formation in exponentially growing MRC-5 cells between IR-A and IR-B, however, there was no difference in micronucleus induction (Fig. 3B). As for 53BP1 focus induction, IR-A significantly induced more 53BP1 foci than IR-B at 1 h post-irradiation in MRC-5 cells for all cell cycle conditions considered in the present study. The same trend appears to be true at 24 and 48 h post-irradiation [25].

The biological effects of secondary particles were easily observed with relatively sensitive endpoints. An immunofluorescent 53BP1 focus assay is a very sensitive method for visualizing DNA damage, and can identify DNA damages induced by mGy of irradiation [26]. A significant difference in the formation of 53BP1 foci was observed in all three cell cycle conditions (Fig. 3A). The micronucleus assay was also used to investigate the effects of secondary particles. In general, IR-A induced a higher frequency of micronuclei than IR-B (Fig. 3B). The trend of micronucleus induction in all three cell cycle conditions was consistent with the trend of 53BP1 foci induction. The LET value of Fe ions is 184.9 KeV/μm and 441.6 keV/μm for 500 and 200 MeV/u, respectively. After shielding that reduces the energy of the Fe ions to 200 MeV/u, only 39% of the particles are still Fe ions; the remainder of the particles are lighter ions and, based on the results of simulated calculation, the average LET value of the shielded beam would be between 0.46 keV/μm and 441.6 keV/μm. In the present study, we showed that the yield of 53BP1 foci and of micronuclei induction for the shielded beam was in general between the yields for 500 and 200 MeV/u Fe ions, in agreement with the prediction based on the LET values for the particles. [25, 27].

We also investigated the secondary effects of three different shielding materials: PMMA, PE and Al (Fig. 4). It is interesting to note that shielding with aluminum appeared to produce lower yield of 53BP1 foci in comparison with PMMA and PE at 1 h post-irradiation. Also noted is the comparison of the induction of micronuclei and 53BP1 between the two cell types. Although the initial, as well as the residual, yield of 53BP1 foci was similar for the two cell types, the frequency of micronuclei was significantly higher in 92-1 cells. Our data indicate that the induction and residual 53BP1 foci may not be a marker for radiosensitivities.

Since the energy of HZE particles in GCR are as high as GeV/u, and unable to be completely blocked by physical methods, another inconvenient element is the LET. Biological effects of ionizing radiation are LET-dependent, and peak at ∼200 keV/μm [28, 29]. The LET of 500 MeV/u iron ions is 184.9 keV/μm, and that of 200 MeV/u is 441.6 keV/μm. Since the secondary particles are lighter than Fe ions, the average LET of the shielded beam would be lower than 441.6 keV/μm, and the biological effects of the shielded beam, as measured by micronuclei, would be higher than for the 200 MeV/u Fe ions, as shown in the present study. For the same reason, the shielded beam would deliver more particles to a cell for the same dose than the 200 MeV/u Fe ions, resulting in a higher yield of 53BP1 foci. The denser cluster of foci induced by the 200 MeV/u Fe ions would also contribute to the lower foci yield. However, particles of other LET values may produce other secondary effects, and require further studies.

## CONCLUSION

In conclusion, we compared the effects of shielded beams with pure beams on cells in different cell cycle conditions, and provided experimental proof that the biological effects of secondary particles produced by the interaction between heavy particles and shielding materials were different from those produced by the primary particles. These findings provide a biological basis for design of space shielding.

## FUNDING

This work was supported by the Research Project with Heavy Ions at NIRS-HIMAC and was also supported by grants of the Major State Basic Research Development Program of China (973 Program, No. 2010CB834201), the Strategic Priority Research Program of the Chinese Academy of Sciences (No. XDA01040411), and the National Natural Science Foundations of China awarded to G.Z. (No. 10979062) and J.W. (Nos U1232125 and 31270895).
